# Functional proteomic analysis reveals the involvement of KIAA1199 in breast cancer growth, motility and invasiveness

**DOI:** 10.1186/1471-2407-14-194

**Published:** 2014-03-15

**Authors:** Mohammad-Saeid Jami, Jinxuan Hou, Miao Liu, Michelle L Varney, Hesham Hassan, Jixin Dong, Liying Geng, Jing Wang, Fang Yu, Xin Huang, Hong Peng, Kai Fu, Yan Li, Rakesh K Singh, Shi-Jian Ding

**Affiliations:** 1 Department of Pathology and Microbiology, University of Nebraska Medical Center, Omaha, NE 68198, USA; 2 Department of Oncology, Zhongnan Hospital of Wuhan University, Wuhan 430071, China; 3 Eppley Cancer Institute, University of Nebraska Medical Center, Omaha, NE 68198, USA; 4 Department of Biostatistics, University of Nebraska Medical Center, Omaha, NE 68198, USA; 5 Biomarker Discovery and Development Laboratory, Sanford-Burnham Medical Research Institute at Lake Nona, Orlando, FL 32827, USA

**Keywords:** Breast cancer, KIAA1199, Quantitative proteomic analysis

## Abstract

**Background:**

*KIAA1199* is a recently identified novel gene that is up-regulated in human cancer with poor survival. Our proteomic study on signaling polarity in chemotactic cells revealed KIAA1199 as a novel protein target that may be involved in cellular chemotaxis and motility. In the present study, we examined the functional significance of KIAA1199 expression in breast cancer growth, motility and invasiveness.

**Methods:**

We validated the previous microarray observation by tissue microarray immunohistochemistry using a TMA slide containing 12 breast tumor tissue cores and 12 corresponding normal tissues. We performed the shRNA-mediated knockdown of KIAA1199 in MDA-MB-231 and HS578T cells to study the role of this protein in cell proliferation, migration and apoptosis *in vitro*. We studied the effects of KIAA1199 knockdown *in vivo* in two groups of mice (n = 5). We carried out the SILAC LC-MS/MS based proteomic studies on the involvement of KIAA1199 in breast cancer.

**Results:**

KIAA1199 mRNA and protein was significantly overexpressed in breast tumor specimens and cell lines as compared with non-neoplastic breast tissues from large-scale microarray and studies of breast cancer cell lines and tumors. To gain deeper insights into the novel role of KIAA1199 in breast cancer, we modulated KIAA1199 expression using shRNA-mediated knockdown in two breast cancer cell lines (MDA-MB-231 and HS578T), expressing higher levels of KIAA1199. The KIAA1199 knockdown cells showed reduced motility and cell proliferation *in vitro*. Moreover, when the knockdown cells were injected into the mammary fat pads of female athymic nude mice, there was a significant decrease in tumor incidence and growth. In addition, quantitative proteomic analysis revealed that knockdown of KIAA1199 in breast cancer (MDA-MB-231) cells affected a broad range of cellular functions including apoptosis, metabolism and cell motility.

**Conclusions:**

Our findings indicate that KIAA1199 may play an important role in breast tumor growth and invasiveness, and that it may represent a novel target for biomarker development and a novel therapeutic target for breast cancer.

## Background

Breast cancer as the most commonly diagnosed and the second leading cause of cancer-related death in women, is responsible for approximately 40,000 deaths in the United States each year [1]. At the time of diagnosis, a majority of patients have metastases to regional and distant sites, which is a major cause of cancer-related mortality [2]. Chemotaxis, cellular migration driven by chemokine gradients, is a critical process involved in tumor invasion and metastasis in various types of cancers including breast cancer [2]. Cell migration is a highly polarized process characterized by protrusion of a leading pseudopodium at the front and establishment of a trailing rear compartment or tail region at the back. Our earlier, comprehensive proteomic analysis of the pseudopodium and cell body in chemotactic cells provided a rich source of information for investigating key signaling pathways and proteins involved in chemotaxis and cancer metastasis [3]. When we compared our pseudopodium proteome dataset with the breast cancer gene expression dataset [4], a protein without a defined function in breast cancer, KIAA1199, caught our attention, as only identified in pseudopodium and highly up-regulated in aggressive breast cancer tissues and cells.

The *KIAA1199* gene which was first discovered to be involved in non-syndromic hearing loss is expressed in a wide range of normal human tissues, with the highest expression level in brain [5]. The *KIAA1199* gene is located on 15q25, where a brain tumor suppressor gene has been mapped [6]. It is highly expressed in three basal type B breast cancer cell lines (HS578T, MDA-MB-231, and BT549) and the expression of this gene is significantly correlated with the invasive ductal carcinoma type of breast cancer [7]. Also, the high expression of KIAA1199 in gastric tumors is associated with a poor prognosis and with lymph node metastasis [8]. These findings are consistent with a recent report which showed that repression of KIAA1199 attenuates Wnt-signaling and decreases the proliferation of colon cancer cells [9]. Other studies have shown that up-regulation of the *KIAA1199* gene is associated with cellular mortality [10] and that the KIAA1199 expression level is significantly elevated upon p53 activation [11]. Based on these observations, we hypothesized that KIAA1199 is a novel regulator of breast cancer growth and aggressiveness.

In this report, we demonstrated the overexpression of KIAA1199 mRNA and protein in breast tumors and invasive cell lines as compared to non-neoplastic tissue and non-invasive cells. Knockdown of KIAA1199 inhibited cell proliferation and motility *in vitro* and tumor incidence and growth *in vivo*. Our comprehensive functional proteomic study to analyze the consequences of KIAA1199 knockdown in the breast cancer cell line MDA-MB-231 demonstrate that KIAA1199 may play an important role in the pathogenesis of breast cancer and that it may represent a novel therapeutic target for breast cancer.

## Methods

### Reagents and cell culture

Fetal bovine serum (FBS), phosphate buffered saline (PBS), Dulbecco’s minimum essential medium (DMEM), penicillin, G418, streptomycin and the rabbit monoclonal anti-cleaved caspase 3 (clone 9H19L2) were purchased from Invitrogen (Gaithersburg, MD). Lysine and Arginine depleted DMEM, McCoy’s 5A medium, Hank’s balanced salt solution (HBSS), depleted FBS, L-[^12^C_6_]arginine, L-[^12^C_6_]lysine, L-[^13^C_6_]arginine, and L-[^13^C_6_]lysine were obtained from Thermo Scientific (Rockford, IL). PGPH1/GFP/NEO shRNA expression vector was obtained from Genepharma (Shanghai, China). Acrylamide, bis, tris base, glycine, ammonium persulphate, PVDF membrane, TEMED, DTT, SDS, urea, thiourea, glycerol, 3-(4,5-dimethylthiazol-2-yl)-2,5-diphenyltetrazolium bromide (MTT), ammonium bicarbonate, DMSO, ECL, bromoplenol blue were purchased from Fisher Scientific (Pittsburgh, PA). Annexin-V-FLUOS Staining Kit was purchased from Roche Applied Science (Mannheim, Germany). The cell culture dish and transwell® with 8.0 μm pore polycarbonate membrane filters were obtained from Corning Corp (Corning, NY). The rabbit polyclonal anti-KIAA1199 antibody, trypsin and trypan blue were obtained from Sigma-Aldrich (St. Louis, MO). Another rabbit polyclonal anti-KIAA1199 antibody was obtained from Protein Tech Group (Chicago, IL). The mouse monoclonal anti-proliferating cell nuclear antigen (PCNA) and rabbit polyclonal anti-alpha-tubulin were respectively purchased from Santa Cruz (CA) and Abcam (MA).

MDA-MB-231 and Hs578T cells (obtained from ATCC (Manassas, VA)) were cultured in DMEM containing 10% FBS, 100 U/ml penicillin and 100 μg/ml streptomycin at 37˚C in an atmosphere containing 5% CO_2_. The SILAC labeling was performed according to the manufacture’s protocol. The lysine and arginine depleted DMEM medium supplemented with L-[^12^C_6_]arginine and L-[^12^C_6_]lysine was used for light condition and the depleted DMEM medium supplemented with L-[^13^C_6_]arginine and L-[^13^C_6_]lysine was used for heavy condition.

### Knockdown of KIAA1199 by shRNA-mediated RNA interference

Four different sets of annealed oligonucleotides specific for the KIAA1199 gene sequence were cloned into the pGPH1/GFP/NEO shRNA expression vector obtained from Genepharma (Shanghai, China). These vector constructs (in addition to an empty vector) were transfected into MDA-MB-231 and Hs578T cells to generate the KIAA1199 knockdown cells (ShA and ShB) and control (ShNC) cells respectively. Since the shRNA plasmids contain the neomycin resistance gene and green fluorescence protein (GFP) expression cassette the transfected cells were selected using 400 μg/ml of G418 (Invitrogen, Carlsbad, MD) and monitored by fluorescent microscopy (Leica, Bannockburn, IL) and flow cytometry.

### Western blot analysis

Western blot analyses were performed on cell lysates prepared from MDA-MB-231 and Hs578T cell lines as described previously [12]. Briefly, triplicate cell cultures were first washed with phosphate buffered saline (PBS, Invitrogen) and then lysed by mixing 1:1 with 2× sodium dodecyl sulphate sample buffer (100 mM Tris–HCl, pH = 6.8, 200 mM DTT, 4% SDS, 20% glycerol and 0.002% bromoplenol blue). Cell lysates were separated by 10% SDS-PAGE. Proteins were transferred to PVDF membranes (Immobilon 0.45 μm, Millipore, USA) and immersed in a blocking solution containing 5% non-fat milk and 0.1% Tween-20 for 1 h. The membranes were washed and incubated with primary antibodies (rabbit polyclonal anti-alpha-tubulin (abcam) at 1:1000 dilution, rabbit polyclonal anti-KIAA1199 (Sigma-Aldrich) at 1:100 dilution, rabbit ployclonal anti-KIAA1199 antibody (Protein Tech Group, Chicago, IL) at 1:800 dilution or rabbit anti-Caspase-3 (8G10) monoclonal antibody (Cell Signaling) at 1:1000 dilution) for 2 h and then with secondary antibodies for 1 h at room temperature. After washing the resulting bands were visualized using the standard ECL procedure, quantified by densitometry and normalized to the corresponding α-tubulin bands.

### mRNA analysis

Total-RNA was extracted from 1×10^7^ cells (cultured in triplicate) using Trizol reagent (Invitrogen,Carlsbad, CA) according to the manufacturer’s instructions. RNA (2-5 μg) was treated with DNAse I (Promega), then reverse transcribed, using 200 U Superscript II (Invitrogen) and 250 ng random primers (Invitrogen), according to the manufacturer’s instructions. The resulting cDNA diluted 1:5 in nuclease-free water and stored in aliquots at −80°C until used. The RT-PCR amplification of KIAA1199 was performed with a denaturation step at 95°C for 10 min, followed by 32 cycles of denaturation at 95°C for 1 min, primer annealing at 56°C for 30 s, and primer extension at 72°C for 30 s. The PCR conditions varied for S100A11 (35 cycles, annealing at 60°C for 30 s, and primer extension at 72°C for 45 s), WASL (28 cycles, annealing at 60°C for 45 s, and primer extension at 72°C for 90 s), PPP1R9B (30 cycles, annealing at 60°C for 30 s, and primer extension at 72°C for 60 s) and GAPDH (30 cycles, annealing at 53°C for 30 s, and primer extension at 72°C for 30 s). Upon completion of the cycling steps, a final extension at 72°C for 5 min was done for all of the reactions and then the reactions were stored at 4°C. The bands obtained after electrophoresis were quantified by densitometry and their intensities were normalized to that provided by the GAPDH (Glyceraldehyde 3-phosphate dehydrogenase) band (relative integral optical density (IOD)) as described before [13]. The average intensity value of the transcripts obtained from the negative control cells were set to 100%. A list of primers is provided in Additional file 1: Table S1.

### Cell motility and migration assay

Wound healing assay was performed to determine cellular motility as described before [14]. Briefly, cells were separately seeded at a density of 5 × 10^5^ cell/well in a 6-well plate (triplicate for knockdown and control cells) and grown to confluence in serum containing DMEM media. The monolayer was scratched using a pipette tip and washed with PBS to remove floating cells and refed with serum containing DMEM media. The wounds were photographed immediately after scratching and again 24 h refeeding. The inhibition in wound closure was qualitatively evaluated.

In order to quantitatively examine the effect of KIAA1199 knockdown in breast cancer cells, we performed trans-well motility assays utilizing 6.5 mm Transwell® with 8.0 μm pore polycarbonate membrane filters (Corning Corp, Corning, NY). Single cell suspensions were seeded onto the upper surface of the filters in supplemental serum free McCoy’s 5A medium. The bottom chamber contained 1.0 ml serum containing media. MTT (3-(4,5-dimethylthiazol-2-yl)-2,5-diphenyltetrazolium bromide) was added and cells were incubated for an additional 3 h. Cells from the top of the transwell chambers were removed using a cotton swab (residual cells). The transwell chambers (migrated cells) and cotton swab containing residual cells were plated in separate well of a 24-well plate containing 400 μl of DMSO. Following 1 h of gentle shaking, 100 μl samples were removed and absorbancy was determined at 570 nm using a microtiter plate reader. The percent migratory activity was calculated as: percent migration = [(A / B) – 1 × 100], where A is the number of migrated cells and B is the number of residual cells*.* Percent migratory activity was compared between different groups. The assay was performed in triplicate.

### Cell proliferation and apoptosis assay

MDA-MB-231 and Hs578T stable cell lines were plated at 2 × 10^3^ cells/well in 96-well plates (triplicate for knockdown and control cells). Following overnight adherence, cells were incubated with serum containing media for various durations. Cell proliferation was determined by MTT (3-(4,5-Dimethylthiazol-2-yl)-2,5-diphenyltetrazolium bromide, a yellow tetrazole) assay. The differences in absorbance were compared in vector control transfected cells and KIAA1199 knockdown cells. To determine the role of KIAA1199 in apoptosis, isogenic variants of MDA-MB-231 and Hs578T stable cell lines were grown in DMEM with 10% FBS. A total of 1×10^6^ cells were washed with PBS (phosphate buffered saline), collected and double-stained for Propidium Iiodide (PI) and Annexin V using the Annexin-V-FLUOS Staining Kit (Roche Applied Science, Mannheim, Germany) according to the manufacturer’s instructions. The frequency of apoptotic cells was analyzed using the FACSCalibur flow cytometer (BD Biosciences, San Jose, CA) with CellQuest Pro software (BD Biosciences).

### Tumor growth assay

Mice were housed and handled according to protocols approved by the University of Nebraska Medical Center Institutional Animal Care and Use Committee. Two groups (n = 5) of female BALB/C nude mice (Charles River, Wilmington, MA), 6–8 weeks of age, housed under pathogen free conditions were used. MDA-MB-231-ShNC and MDA-MB-231-ShB cell monolayers were trypsinized and washed with Hank’s balanced salt solution (HBSS) 3 times and counted using trypan blue (Sigma) exclusion dye. Single cell suspensions of 1x10^6^ cells (>95% viability) in 100 μL were injected into the mammary fat pad. Twice a week tumor size was measured using digital calipers (Fisher Scientific, Pittsburgh, PA). Tumor volume was calculated according to the formula Volume = W^2^ × L/2, where W = short diameter, and L = long diameter. Mice were euthanized and primary tumors were removed and processed by formalin fixation with subsequent embedding in paraffin for immunohistochemistry.

### Immunohistochemical analysis

IHC analysis was performed as described previously [15] using the rabbit polyclonal anti-KIAA1199 (Sigma-Aldrich; 1:10 dilution), the rabbit monoclonal anti-cleaved caspase 3 (CASP3; Invitrogen; 1:500 dilution) and the mouse monoclonal anti-proliferating cell nuclear antigen (PCNA; Santa Cruz, CA; 1:40 dilution) as primary antibodies. Tumor sections were deparaffinized by incubation in EZ-Dewax (BioGenex Laboratories Inc, San Ramon, CA) and rinsed in distilled water to remove residual EZ-Dewax. Following nonspecific blocking for 30 min, sections were incubated with primary antibodies overnight at 4°C. Sections were then washed and subsequently incubated at room temperature with the respective biotinylated secondary antibodies (1:500 in PBS) for 45 min. Immunoreactivity was visualized by incubating the avidin-biotin complex with diaminobenzidine tetrahydrochloride substrate (Vector Laboratories, Burlingame, CA). The sections were observed microscopically (Nikon, Melville, NY) using 5 × 5 reticle grid (Klarmann Rulings, Litchfield, NH) and stained cells and vessels were identified. The slides were lightly counterstained with Harris hematoxylin and viewed under a light microscope.

The breast cancer TMA slide (catalog number A712(12) and A712(13)) was purchased from AccuMax (Seoul, Korea). A human kidney tissue was used as positive control. The slide was processed for IHC detection of KIAA1199 expression with a polyclonal anti-KIAA1199 primary antibody (1:10 dilution; SigmaAldrich). An iSan Coreo slide scanner (Ventana Medical Systems, AR) was used to scan the slide at 40× and the resulting images were analyzed by Metamorph Imaging Software (Molecular Devices, CA) to determine the intensity of immunostaining. Immunostaining index (arbitrary unit) was calculated by considering the level of immunostaining intensity and the area with KIAA1199 positivity.

### Quantitative proteomic analysis

MDA-MB-231-ShNC (cultured in light medium) and MDA-MB-231-ShB Cells (cultured in heavy medium) were grown in doublet SILAC conditions and the proteomic samples were prepared as previously described [16]. Briefly, MDA-MB-231-ShNC and MDA-MB-231-ShB cells were seeded at 20–30% confluence and harvested when cell density reached 90%. After 10 passages, heavy (Arg6, Lys6) labeled MDA-MB-231-ShB and MDA-MB-231-ShNC cells (Light) were harvested separately in 7 M urea, 2 M thiourea and 50 mM ammonium bicarbonate. Equal amounts of protein were combined from each condition. Following tryptic digestion and chromatography separation via strong cation exchange (SCX), a total of 21 fractions of peptide mixtures were subjected to C18 reverse-phase liquid chromatography (Eksigent, Dublin, CA) coupled online to an LTQ-Orbitrap mass spectrometer (Thermo Scientific, Bremen, Germany). Two biological replicates were performed. The MS data were analyzed using the UNiquant software pipeline [16]. Briefly, DeconMSn (http://omics.pnl.gov/software/) was used to determine and refine the monoisotopic mass and charge state of parent ions from the LTQ-Orbitrap raw data, and to create a peak list of these ions in .mgf format. The peak list contained the fragment information such as the MS/MS spectra, refined precursor ion and charge state. DtaRefinery (http://omics.pnl.gov/software/) was used to improve mass measurement errors for parent ions of tandem MS/MS data by modeling systematic errors based on putative peptide identifications using the algorithm as described [16]. A script written in Python (programming language) was used to automate the process of generating .mgf files from raw data using DeconMSn and DtaRefinery. The resulting .mgf file was submitted to Mascot (version 2.2, Matrix Science, London, U.K.) database searching against (i) a concatenated database containing 73,928 proteins from international protein index (IPI) database (version 3.52), (ii) the commonly observed 262 contaminants (forward database), and (iii) the reversed sequences of all proteins (reverse database). Carbamidomethylation was set as the fixed modification and oxidation of methionine was set as the variable modification. The initial mass deviation tolerance of precursor ion was set to 10 ppm and fragment ion tolerance was set to 0.5 Da. A maximum of 2 missed cleavages were allowed in peptide identification. Identified peptides were sorted by a descending order of Quality of Peptide Identification (QPI) which is defined by the Mascot peptide identification score (a minimum of 10) divided by the square root of the precursor ion mass error. A cutoff of QPI was applied to ensure a total false discovery rate (FDR) for peptide identification < 0.01 evaluated by reverse database approach [16].

### Statistical analysis

*In vivo* data analysis was performed using the Mann–Whitney *U*-test for significance. For the quantitative analysis of differentially expressed proteins identified by LC-MS/MS, a mixed-effects model with random effects from the two experimental runs was fit to the log2 of the protein fold changes to test whether the log2 of protein fold change was significantly different from zero. Note that a differentially expressed protein is expected to have a non-zero log2 fold change. The p-value was calculated and further corrected by the Benjamini-Hochberg (BH) procedure [17] to control the false discovery rate to be no more than 0.05. A protein with a BH corrected *p*-value equal-to-or-less-than 0.05 was considered to be statistically significant. For the TMA analysis immunostaining index was tested using the paired t-test to determine the significance of difference between the carcinoma and non-neoplastic cores. The TMA results were reviewed by three independent pathologists.

### Ethics statement

All procedures performed *in vivo* tumor growth and metastasis studies were in accordance with institutional guidelines and approved by the University of Nebraska Medical Center Institutional Animal Care and Use Committee.

## Results

### Expression of KIAA1199 in breast cancer specimens

**Figure 1:**
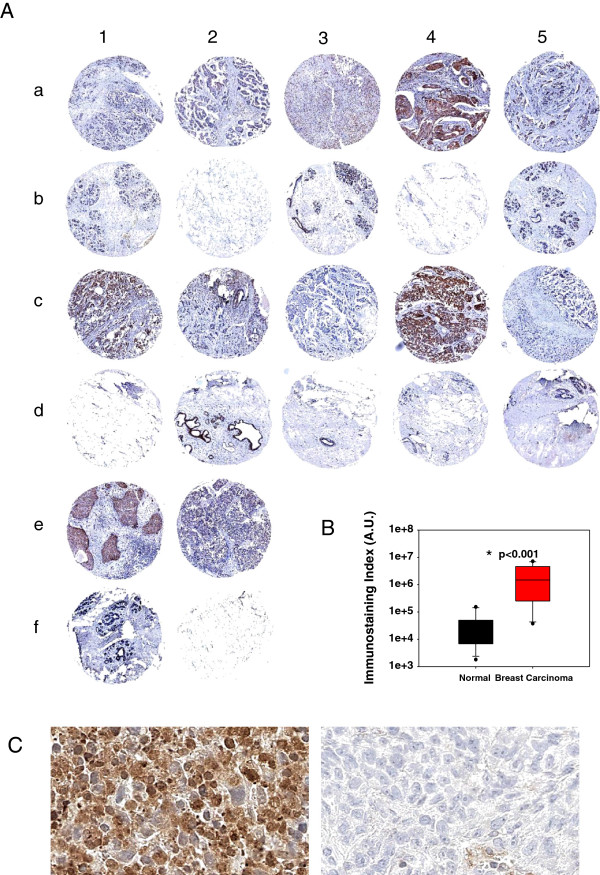
**KIAA1199 expression in breast cancer tissues. A)** The TMA slide (×4) contained 12 tumor tissue cores (rows a, c and e) and 12 corresponding normal tissues (rows b, d and f) were immunostained with anti-KIAA1199 antibody. **B)** Evaluation of KIAA1199 expression by calculation of immunostaining index using the Metamorph software; the box plot shows a significant difference in KIAA1199 expression between breast carcinoma tissues and the corresponding non-neoplastic normal tissues. **C)** Representative magnification (×400) of KIAA1199 immunostaining in two cores (c4 vs. d4) shows the cytosolic localization of this protein.

In order to assess the clinical relevance of KIAA1199 in breast cancer we performed a bioinformatics study of a large database of microarray data from cancer experiments available at the Oncomine website (http://www.oncomine.org). We observed the overexpression of KIAA1199 mRNA in breast tumor tissues (see Discussion) as compared to non-neoplastic tissue (Table 1). We performed a tissue microarray (TMA) analysis to examine the KIAA1199 protein expression level in breast carcinoma and normal tissues (Table 2). As shown in the Additional file 2: Figure S1 a human kidney tissue was used as positive (cells in tubules) and negative (cells in glomeruli) control for immunohistochemical staining (according to the human protein atlas at http://www.proteinatlas.org KIAA1199 has the highest expression level in renal tubules). Figure 1 illustrates the cytosolic localization of KIAA1199 and results of immunohistochemical staining of a TMA slide containing 12 breast tumor tissue cores (rows a, c and e) and 12 corresponding normal tissues (rows b, d and f). We quantified and evaluated the KIAA1199 protein expression by analyzing the intensity of immunostatining and positive areas percentage in each core image using the Metamorph software (Zeiss). We observed a 14.66 fold overexpression of KIAA1199 protein in breast tumor tissues (t-test, p = 0.025) compared to non-neoplastic breast tissues (Figure 1).

**Table 1:** Microarray studies in different breast cancer types

**Reporter**	**Cancer type**	**Breast samples**	**Tumor samples**	**t-Test**	**p-Value**	**Fold change**
TCGA^a^	Invasive Breast Carcinoma	61	76	14.019	3.39E-28	9.094
TCGA	Invasive Ductal Breast Carcinoma	61	392	19.021	1.71E-36	8.233
TCGA	Invasive Lobular Breast Carcinoma	61	36	8.501	7.32-12	5.527
Gluck *et al.*^*b*^	Invasive Breast Carcinoma	4	154	9.603	2.48E-7	2.926
Richardson *et al.*^*c*^	Ductal Breast Carcinoma	7	40	6.564	1.06E-6	4.125

^a^)The Cancer Genome Atlas data was obtained from the Oncomine website.

^b^)See Reference [19].

^c^)See Reference [20].

Several studies show the overexpression of KIAA1199 in breast carcinoma comparing to normal breast tissues.

**Table 2:** Details about each core on the TMA slide

**Core**	**Breast cancer type**	**Sex**	**Age**	**Tissue area**	**Threshold (%)**	Log2^*^
a1	Phyllodes Tumor	F	45	271599	0.15	−2.72
b1	Non-neoplastic	F	45	254568	0.12	−3.04
a2	Infiltrating Ductal Carcinoma	F	58	332807	1	0
b2	Non-neoplastic	F	58	191591	0	−9.32
a3	Invasive Lobular Carcinoma	F	51	326860	3.12	1.64
b3	Non-neoplastic	F	51	247173	0.64	−0.65
a4	Infiltrating Ductal Carcinoma	F	66	332029	18.21	4.19
b4	Non-neoplastic	F	66	143861	0.01	−6.68
a5	Infiltrating Ductal Carcinoma	F	54	373279	0.54	−0.88
b5	Non-neoplastic	F	54	277105	0.16	−2.65
c1	Infiltrating Ductal Carcinoma	F	55	340233	8.12	3.02
d1	Non-neoplastic	F	55	83421	0	−8.7
c2	Infiltrating Ductal Carcinoma	F	63	273915	1.44	0.53
d2	Non-neoplastic	F	63	270038	2.87	1.52
c3	Atypical Medullary Carcinoma	M	72	306756	0.02	−5.88
d3	Non-neoplastic	M	72	195427	0.07	−3.84
c4	Infiltrating Ductal Carcinoma	F	64	358767	23.08	4.53
d4	Non-neoplastic	F	64	215357	0	−11.07
c5	Atypical Medullary Carcinoma	F	49	253762	0.02	−5.45
d5	Non-neoplastic	F	49	304971	0.18	−2.51
e1	Infiltrating Ductal Carcinoma	F	38	355620	7.01	2.81
f1	Non-neoplastic	F	38	260062	0.1	−3.38
e2	Infiltrating Ductal Carcinoma	F	41	381085	0.87	−0.2
f2	Non-neoplastic	F	41	30471	0.19	−2.37

*p-value = 0.025, T-test = 2.581.

The IHC staining of 12 tumor and 12 non-neoplastic tissues cores on the TMA slide (Figure 1) was evaluated based on log2 of%Threshold. The T-test showed the significant difference of KIAA1199 expression between non-neoplastic breast tissues and breast tumor tissues (overall 14.66 fold overexpression of KIAA1199 in tumor tissues).

### Knockdown of KIAA1199 in breast cancer cell lines

The construction of the silencing vector pGPH1/GFP/NEO is shown in Additional file 3: Figure S2. Two different sets of annealed oligonucleotides (ShA and ShB) were used to knockdown the KIAA1199 gene in both MDA-MB-231 and Hs578T cells. We evaluated the efficiency of knockdown through both RT-PCR and Western blotting approaches in triplicate. As shown in the Additional file 3: Figure S2, we observed an average of 86% and 92% decrease in the level of KIAA1199 transcription in MDA-MB-231-ShA and MDA-MB-231-ShB cells, respectively. The attenuation rate in Hs578T cell line was 63% and 90% for Hs578T-ShA and Hs578T-ShB cells. Reduction of KIAA1199 protein expression was 86% for MDA-MB-231-ShA cells and 97% for MDA-MB-231-ShB cells; similarly we observed 22% and 85% decrease in Hs578T-ShA and Hs578T-ShB cells. These data suggest that ShB construct was more effective in KIAA1199 knockdown in both breast cancer cell lines.

### KIAA1199 knockdown inhibits *in vitro* cell proliferation and migration and enhances apoptosis

**Figure 2:**
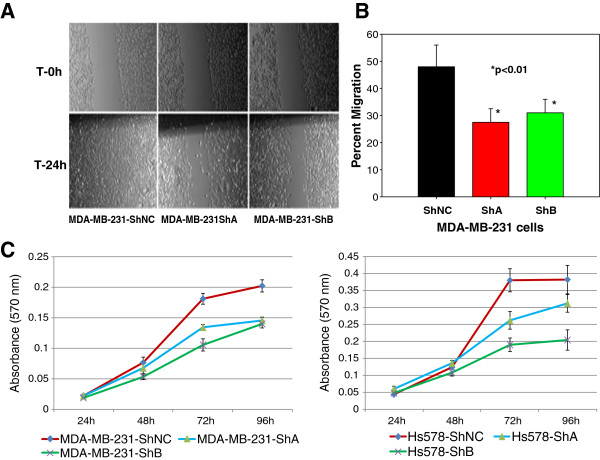
**Knockdown of KIAA1199 inhibits cell migration and proliferation *in vitro*. A)** The wound-healing assay shows significantly lower cell motility in the KIAA1199 knockdown cells (MDA-MB-231-ShA and MDA-MB-231-ShB) compared to the negative controls. **B)** Trans-well assay shows a decrease in the cell migration rate (migrated/total) for the KIAA1199 knockdown cells (the experiment was performed in three biological replicates). **C)** The MTT assay demonstrates that both MDA-MB-231 and Hs578T knockdown cells have significantly lower proliferation levels at 72 and 96 h of culture (the experiment was performed in three biological replicates).

A wound-healing assay qualitatively showed that cell motility was impaired in MDA-MB-231-ShA and MDA-MB-231-ShB cells as compared to the negative control (MDA-MB-231-ShNC) cells (Figure 2A). Similarly, the transwell migration assay (Figure 2B) showed an average of 44% inhibition of cell migration for MDA-MB-231-ShA cells and 31% inhibition for MDA-MB-231-ShB cells as compared to control MDA-MB-231-NC cells (the experiment was performed in three biological replicates). These data suggest that knockdown of KIAA1199 significantly inhibits the cell motility in MDA-MB-231 cells. However, no significant change in cell motility was observed after KIAA1199 knockdown in Hs578T cells (data not shown).

Next, we examined whether KIAA1199 knockdown modulated breast cancer cell proliferation. KIAA1199 knockdown in both MDA-MB-231 and Hs578T cells (the experiment was performed in three biological replicates) significantly inhibited the cell proliferation (Figure 2C) as compared to the vector control transfected cells (t-test, *P <* 0.05).

**Figure 3:**
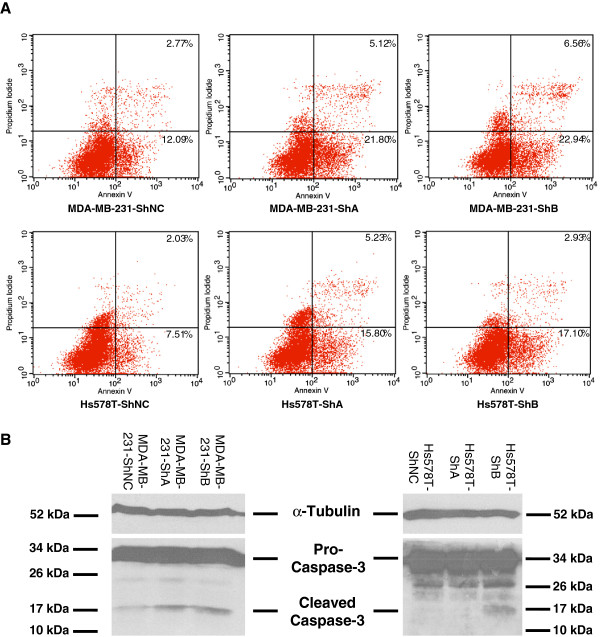
**KIAA1199 Knockdown enhanced apoptosis *in vitro*. A)** Flow cytometry analysis shows a large increase in the percentage of cells programmed for apoptosis in MDA-MB-231-ShA, MDA-MB-231-ShB, Hs578T-ShA and Hs578T-ShB cells comparing to the corresponding negative controls. **B)** Confirmation of the results of Flow cytometry analysis by Western blot (single experiment). Caspase-3 activation is detected in Western blots by the presence of cleavage fragments. The antibody detects both pro (full-length) and active (cleaved) protein. The increased representation of cleaved caspase-3 in KIAA1199 knockdown cells compared to the control cells is qualitatively shown in MDA-MB-231 (left panel) and Hs578T (right panel) cells.

In order to study the effect of KIAA1199 knockdown on apoptosis, we performed flow cytometric analysis using AnnexinV^+^ (early apoptosis marker) and AnnexinV^+^/PI^+^ (late apoptosis) cells. We observed higher frequency of cells programmed for both early and late phases of apoptosis in KIAA1199 knockdown cells as compared to vector controls (Figure 3A). We observed an average of 1.72 and 1.94 fold increase in early apoptosis rate in MDA-MB-231-ShA and MDA-MB-231-ShB cells comparing to negative controls cells. The increase of late apoptosis rate for these cells was 1.82 and 2.36 fold respectively. In addition, similar results were observed in Hs578T cell line; Hs578T-ShA and Hs578T-ShB cells showed 2.19 and 2.26 fold increase in the rate of early apoptosis. KIAA1199 knockdown cells also showed higher (2.61 and 1.45 fold) rate of late apoptosis (Figure 3A).

To further confirm the effect of KIAA1199 knockdown on apoptosis, we performed Western blot analysis of caspase-3 using the rabbit anti Caspase-3 (8G10) monoclonal antibody (Cell Signaling) which detects both pro-caspase-3 and cleaved caspase-3. As shown in Figure 3B, we observed an overrepresentation of cleaved caspase-3 in KIAA1199 knockdown cells compared to control cells.

Together these data suggest that KIAA1199 knockdown inhibited cellular migration and proliferation and enhanced apoptosis. Since the MDA-MB-231-ShB seemed to be more efficiently affected during the KIAA1199 we choose to use this cell line together with MDA-MB-231-ShNC for further *in vivo* studies and proteomic analyses.

### KIAA1199 knockdown inhibits tumor incidence/growth and cell proliferation

**Figure 4:**
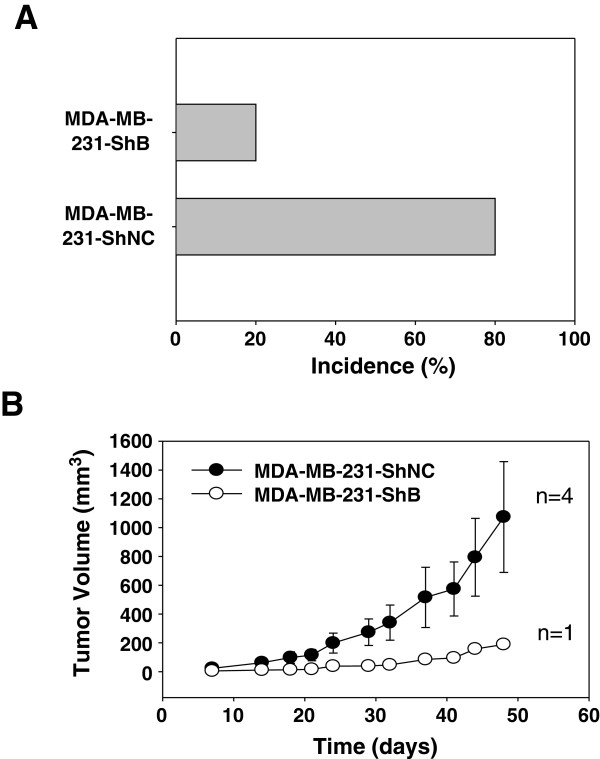
**KIAA1199 Knockdown inhibited tumorigenicity, growth and neovascularization. A)** The relative tumor incidence in MDA-MB-231-ShNC and MDA-MB-231-ShB cell bearing mice; The MDA-MB-231-ShNC (control) and MDA-MB-231-ShB cells were implanted into the mammary fat pads of two groups of nude mice (n = 5). Four of the MDA-MB-231-ShNC and one of the MDA-MB-231-ShB implanted mice developed tumors. **B)** Tumor growth diagram for MDA-MB-231-ShNC and MDA-MB-231-ShB injected mice.

**Figure 5:**
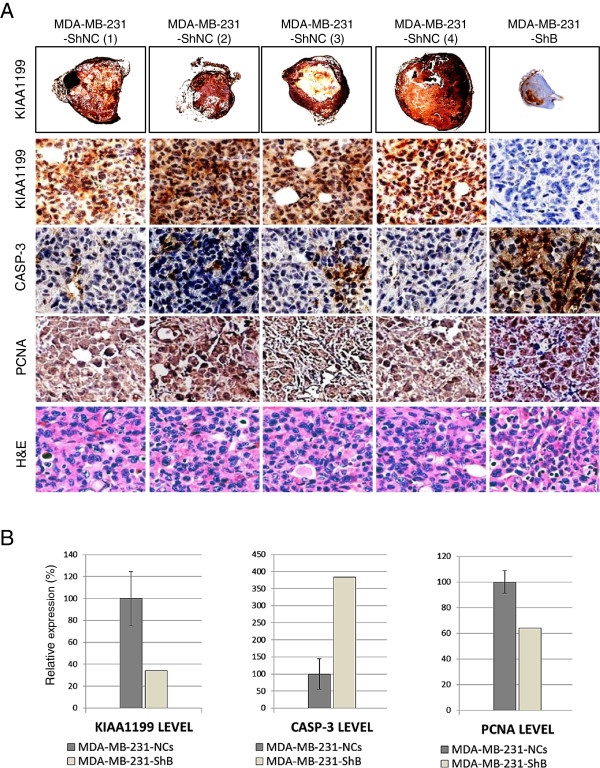
**Immunohistochemical studies. A)** Very low KIAA1199 immunostaining (first row) in MDA-MB-231-ShB tumor comparing to the controls (×4). Representative illustration of immunohistochemical studies (×100 magnifications) shows the higher expression level of KIAA1199 (brown staining cells in the second row), lower apoptosis activity (CASP3, third row) and higher proliferation activity (PCNA, fourth row) than the MDA-MB-231-ShB tumor. **B)** Evaluation of the expression of protein markers by calculation of immunostaining index using the Metamorph software; graphs from left to right show the relative expression of KIAA1199, CASP3 and PCNA in control versus KIAA1199 knockdown tumor sections.

To determine whether KIAA1199 depletion modulates tumor growth, we implanted the MDA-MB-231-ShNC (control) and MDA-MB-231-ShB cells into the mammary fat pads of nude mice (n = 5). We observed significant reduction in tumor incidence following KIAA1199 knockdown (Figure 4A). Four of the MDA-MB-231-ShNC and one of the MDA-MB-231-ShB implanted mice developed tumors. In addition, we observed a significant inhibition in the tumor growth (Figure 4B) in mice bearing the MDA-MB-231-ShB cells as compared to MDA-MB-231-ShNC. We validated the levels of KIAA1199 in tumors using immunohistochemistry. MDA-MB-231-ShNC tumors showed intense KIAA1199 expression whereas MDA-MB-231-ShB tumors showed very little or no immunostaining for KIAA1199 (Figure 5). Moreover, the results showed the cytosolic localization of KIAA1199 protein. Interestingly, several local metastatic foci, expressing even higher levels of KIAA1199, appeared in the fat pads adjacent to the MDA-MB-231-ShNC tumors. These data demonstrate that knockdown of KIAA1199 inhibited MDA-MB-231 tumorigenesis and growth *in vivo*.

Next we examined whether KIAA1199 knockdown modulates *in situ* phenotypes associated with tumor growth and aggressiveness using immunohistochemical analysis of tumors derived from MDA-MB-231-ShNC and MDA-MB-231-ShB cells. The expression level of cleaved caspase 3 (CASP3) protein (the apoptosis initiation marker) is increased in the KIAA1199 knockdown tumors (Figure 5). Moreover, analysis of *in situ* cell proliferation using anti-PCNA antibody demonstrated the inhibition of malignant cell proliferation in the MDA-MB-231-ShB tumor compared to the MDA-MB-231-ShNC tumors (Figure 5). Together these data demonstrate that knockdown of KIAA1199 inhibited *in situ* cell proliferation and enhanced apoptosis.

### Quantitative proteomic analysis of MDA-MB-231-ShNC and MDA-MB-231-ShB cells

**Figure 6:**
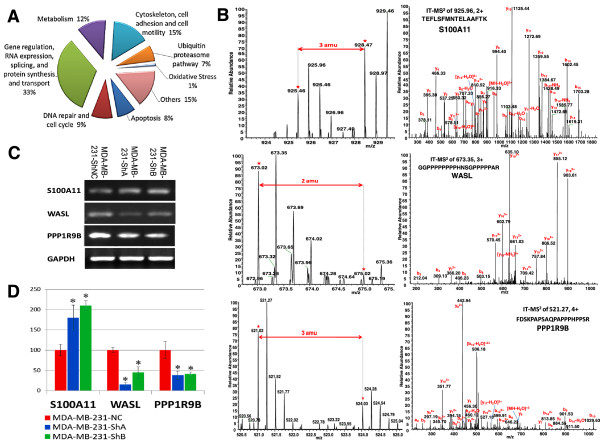
**Representative data from the proteomics study on MDA-MB-231 cells after knockdown of KIAA1199. A)** Differentially expressed proteins were classified based on their Gene Ontology (GO) and function. **B)** MS and MS2 spectra from one up-regulated protein (S100A11) and two down-regulated proteins (WASL and PPP1R9B). The left-hand panels show MS spectra, where the red *symbols show intensities of the monoisotopic peak for the light and heavy SILAC labeled peptides. The right-hand panels show the MS2 spectra corresponding to the peptide with the most intense signal of the pair. **C)** Validation of differential expression of S100A11, WASL and PPP1R9B genes using semi-quantitative RT-PCR analysis in triplicate. **D)** The relative expression of the bands is shown as bars (*p < 0.01). Intensities were normalized to GAPDH band. The average intensity values of the transcripts obtained from the MDA-MB-231-ShNC cells were set to 100%.

Expression of a variety of proteins was affected by KIAA1199 knockdown. These expression changes were characterized through quantitative proteomics (Figure 6). A total of 6,543 unique peptides corresponding to 1,574 proteins were identified (FDR < 0.01) and quantified in the mixture of proteins taken from MDA-MB-231-ShNC (light medium) and MDA-MB-231-ShB (heavy medium) cells (Additional file 4: Table S2) by the UNiquant software pipeline [16]. Although the SILAC based proteomic study was limited to MDA-MB-231-ShNC and MDA-MB-231-ShB cells, the experiment was performed in two independent biological replicates to increase confidence. Total numbers of 1217 and 1404 proteins were identified in replicate 1 and 2 respectively. Among them, 91 proteins were differentially expressed in both replicate experiments (p < 0.05). Using the Kyoto Encyclopedia of Genes and Genomes (KEGG) and the Uniprot Database, the differentially expressed proteins were classified into eight major categories based on their biological roles and their Gene Ontology (GO) (see Figure 6A). Figure 6B shows the results for representative peptides associated with three of the differentially expressed proteins. Our SILAC-based LC-MS/MS study showed the average up-regulation to 1.85 fold for protein S100A11, down-regulation to 0.10 fold for WASL and 0.25 fold for PPP1R9B. In order to validate the protein level alteration we performed the semi-quantitative RT-PCR as a standard method to evaluate the transcription level of these proteins [13]. We observed the average of 1.75 and 2.1 fold over-expression of S100A11 in mRNA level in MDA-MB-231-ShA and MDA-MB-231-ShB respectively. Also the transcription level of WASL/PPP1R9B was decreased to 0.14/0.38 and 0.46/0.43 fold in MDA-MB-231-ShA and MDA-MB-231-ShB respectively. These findings showed the accuracy of normalization method used by the UNiquant software pipeline and validated the mass spectral observations (Figure 6C and D). Further data describing the protein changes are detailed in Additional file 4: Table S2 and summarized in Table 3. As shown in Figure 6A, the functions of proteins differentially expressed between MDA-MB-231-ShNC and MDA-MB-231-ShB cells can be assigned to eight categories including Apoptosis, DNA repair and cell cycle, Gene expression and regulation, Cytoskeleton, cell adhesion and motility, Ubiquitin proteasome pathway, Metabolism, Oxidative stress and other proteins. This data suggest that KIAA1199 may affect a broad range of cellular functions.

**Table 3:** Functional categories of proteins differentially expressed in MDA-MB-231-ShB cells compared to MDA-MB-231-ShNC cells

**Functional categories**	**Protein names**
Apoptosis [n = 7, 8%]	BAX (1.50, 4.53E-02), FADD (1.63, 2.17E-02), DIO-1 (25.39, 5.91E-55), AKAP95 (2.19, 4.12E-06), PGRMC1 (0.62, 0.01), GNAS (0.53, 3.30E-4), TFG (0.59, 6.19E-03)
DNA repair and cell cycle [n = 8, 9%]	SMC1A (1.62, 4.66E-02), ANAPC10 (0.11, 4.30E-44), PPP1CB (0.64, 3.35E-2), PPP2R1A (0.46, 4.1E-06), CRABP2 (4.24, 5.69E-20), C10orf78 (2.21, 3.14E-06), NXN (0.60, 0.01), TK1 (0.61, 8.50E-03)
Gene regulation, RNA expression, mRNA splicing, and protein synthesis and transport [n = 30, 33%]	RBBP4 (1.68, 2.18E-02), WDR5 (8.98, 2.37E-24), ZNF259 (1.52, 4.84E-02), SFRS5 (1.60, 2.16E-02), STAU1 (1.63, 1.30E-02), RPL37A (1.53, 0.04), EIF2S2 (1.57, 0.03), TMED2 (1.56, 2.98E-02), KIAA0521 (1.77, 2.00E-03), SRP14 (2.28, 3.14E-05), HNRPA1L-2 (7.61, 7.77E-38), SRP72 (0.26, 2.07E-17), RGPD5 (0.36, 5.44E-10), PQBP1 (0.44, 2.57E-06), TERF2IP (0.47, 8.95E-06), SEC23B (0.49, 4.08E-05), SUPT5H (0.51, 1.78E-04), FUBP1 (0.52, 2.79E-04), PPP1R14B (0.52, 2.79E04), CPSF3 (0.57, 2.75E-03), HMGA1 (0.58, 5.70E-03), RPS15 (0.60, 6.49E-03), KIAA1150 (0.60, 2.75E-03), ELAC2 (0.61, 9.85E-03), FARSA (0.62, 0.01), SNRNP70 (0.63, 0.02), BASP1 (0.64, 0.03), KIAA0324 (0.65, 0.04), PRPF4 (0.65, 0.48), MAGED2 (0.65, 0.02)
Metabolism [n = 11, 12%]	ATP5C1 (1.62, 0.02), PGLS (0.59, 4.34E-03), PGAM4 (0.38, 6.95E-10), ACAA1 (0.03, 6.80E-104), ACOT2 (0.37, 8.07E-10), USMG5 (1.55, 3.69E-02), GCDH (1.69, 5.90E-03), ALDH9A1 (6,60, 5.67E-33), RRM2 (0.47, 1.18E-06), AK1 (0.49, 1.44E-10), VAT1 (0.57, 3.66E-03)
Cytoskeleton, cell adhesion and cell motility [n = 14, 15%]	S100A11 (1.82, 1.13E-03), TACC3 (0.58, 4.54E-03), WASL (0.10, 3.76E-48), PPP1R9B (0.25, 1.40E-18), TNXB (0.09, 2.43E-52), SEPT9 (0.57, 1.76E-03), NCKIPSD (1.54, 3.95E-02), ACTR3 (11.59, 1.39E-51), LUM (0.07, 7.40E-59), KIAA0345 (0.23, 6.31E20), THBS1 (0.41, 5.28E-09), ARHGEF2 (0.43, 8.89E-07), ZYX (0.43, 1E-06), SDCBP (0.53, 5.15E-04)
Ubiquitin proteasome pathway [n = 6, 7%]	UBE2V1 (0.63, 2.59E-02), ZFP91 (0.53, 3.87E-04), UBE2C (0.49, 3.04E-05), UBE2L3 (0.55, 3.88E-04), UBE2K (0.56, 1.61E-03), KIAA0439 (0.65, 0.03)
Oxidative Stress [n = 1, 1%]	DJ-1 (2.01, 7.10E-04)
Others [n = 14, 15%]	ACP1 (1.52, 4.72E-02), CYR61 (1.56, 3.49E-02), HBA1 (0.02, 7.30E-102), LTF (0.02, 3.80E-102), HBE1 (0.11, 2.89E-42), ALB (0.13, 2.21E-38), CHCHD2 (0.37, 3.94E-10), C19orf43 (0.39, 6,69E-09), CCDC86 (0.40, 4.27E-08), COX17 (0.45, 5.23E-06), C1orf122 (0.46, 6.59E-06), ZC3H18 (0.46, 8.95E-06), TXLNA (0.49, 9.02E-06), C11orf84 (0.52, 3.18E-05)

A total number of 91 differentially expressed proteins were classified by their characteristics and broad functional criteria. The number and the approximate percentage of proteins in each category are shown in brackets. Fold change greater than 1 means that proteins were up-regulated in MDA-MB-231-ShB cells and vice versa.

## Discussion

In order to identify new biomarkers for the improvement of new diagnosis strategies and targeted therapy, it is essential to better understand breast cancer biology and the molecular profiles that will respond to targeted treatment. Molecular markers such as progesterone receptor, estrogen receptor, and ErbB2 have been associated with the five major subtypes of breast cancer: luminal A, luminal B, ErbB2+/ER-, basal-like, and normal breast-like [18]. However, molecular pathways involved in incidence, progression and clinical outcomes remain elusive.

Several microarray based expression studies have previously shown the overexpression of KIAA1199 in breast cancer (Table 1). The results of a recent study from The Cancer Genome Atlas (TCGA) on 593 samples shows 9.094 fold (p = 3.39E-28) overexpression in invasive breast carcinoma, 8.233 fold (p = 1.71E-36) in invasive ductal breast carcinoma and 5.527 fold (p = 7.32E-12) in invasive lobular breast carcinoma compared to corresponding normal breast tissues. Another comparison between invasive breast carcinoma and normal tissue in 158 samples by Gluck and co-workers showed a 2.926 fold (p = 2.48E-7) overexpression of KIAA1199 in invasive breast carcinoma [19]. Furthermore, Richardson and co-workers have reported a 4.125 (p = 1.06E-6) fold overexpression of KIAA1199 in ductal breast carcinoma [20]. In addition to these data, our immunohistochemical study on clinical breast cancer specimens showed 14.66 fold (p = 0.025) overexpression of this protein.

Based on these findings, we examined the role of KIAA1199 in the MDA-MB-231 and Hs578T breast cancer cell lines using two sets of shRNA-mediated knockdown cells for each cell line. We observed that knockdown of KIAA1199 enhanced apoptosis and inhibited cell proliferation and survival in both cell lines *in vitro*. Additionally, using immunohistochemical staining against cleaved caspase-3 (CASP3) and PCNA we respectively confirmed the apoptosis enhancement and inhibition of cell proliferation *in vivo*.

Interestingly, our proteomic study showed that while the negative control cells expressed higher levels of the apoptosis inhibitors, several proteins involved in apoptosis were overrepresented in the knockdown cells justifying the higher apoptotic activity we observed *in vitro* and *in vivo*. For instance the apoptosis regulator BAX which promotes programmed cell death after binding to, and antagonizing the apoptosis repressor BCL2 is up-regulated. BAX also accelerates the activation of CASP3, and thereby promotes apoptosis [21-24]. In addition, we observed the up-regulation of FADD (FAS-Associated Death Domain protein) which is another apoptotic adaptor molecule. FADD bridges the death receptors (e.g. Fas-receptor) to the death-inducing signaling complex (DISC) and activates caspase-8. Active caspase-8 initiates a cascade of caspases which mediate apoptosis [25]. Another example is a large increase in the expression of DIO-1 (death inducer-obliterator-1) that translocates to the nucleus and activates apoptosis in cell culture [26]. KIAA1199 knockdown also led to up-regulation of A-kinase anchor protein 8 (AKAP95) that is a potential carrier protein for active caspase 3, carrying it from the cytoplasm into the nuclei in apoptotic cells and is involved in the process of apoptotic nuclear morphological change [27].

It is noteworthy that we found progesterone (P4) receptor membrane component-1 (PGRMC1) down-regulated upon KIAA1199 knockdown. This protein promotes cell survival in human cancer after chemotherapy [28]. PGRMC1 was reported to be over-expressed in breast tumors and other cancer cell lines [29].

It is known that high expression of BAX is associated with improved chemotherapy responsiveness [30] whereas PGRMC1 has a negative impact on chemotherapy by promoting the survival of treated cancer cells [28]. This knowledge plus the fact that KIAA1199 knockdown alters the expression level of these proteins, suggests that KIAA1199 depletion may potentially improve cellular response to chemotherapy.

Our wound healing and transwell cell motility assays showed lower motility in the MDA-MB-231-ShB cells. These findings can be explained by the observation of altered levels of proteins involved in cellular shape change, filopodia extension, nuclear migration and adhesion inhibition in the knockdown cells. We observed the up-regulation of S100A11 protein which functions in tubulin polymerization, motility, and tumor invasion [31] and down-regulation of the transforming acidic coiled-coil-containing protein 3 (TACC3). The latter plays a role in the microtubule-dependent coupling of the nucleus and the centrosome, and it has been demonstrated to be over-expressed in various cancer cell lines [32]. Furthermore, TACC3 depletion has been reported to strongly sensitize cells to chemotherapy [33], therefore KIAA1199 depletion can also potentially affect the cellular response to chemotherapy via TACC3.

Neural Wiskott-Aldrich syndrome protein (WASL) is dramatically down-regulated (0.10 fold) in the KIAA1199 knockdown cells. WASL activates the Arp2/3 complex required for the extension of lamellipodia and filopodia during cell movement [34]. Another down-regulated protein is Neurabin-2 (PPP1R9B) which binds along the sides of F-actin and plays a role in linking the actin cytoskeleton to the plasma membrane at the synaptic junction. PPP1R9B therefore might be involved in cell shape change and migration [35]. A member of the tenascin protein family, the glycoprotein tenascin X (TNXB) is also dramatically down-regulated in the KIAA1199 knockdown cells. As opposed to fibronectin which is adhesive, the tenascins have anti-adhesive effects. TNXB mediates interactions between cells and the extracellular matrix and may support the growth of epithelial tumors [36]. Overall, these findings suggest that KIAA1199 may be involved in determination of cellular morphology and motility.

However, unlike in MDA-MB-231-ShB cells the cell motility was not affected in Hs578Tcell after KIAA1199 knockdown. Although both of these cell lines belong to basal type B breast cancer, MDA-MB-231 cells was originated from invasive ductal carcinomas (IDC) whilst Hs578TT cells originated from a breast carcinosarcoma, and they highly differ in migration and invasion capability [37]. These data suggest discrete cell migratory mechanisms in these cell lines in which KIAA1199 may or may not participate.

In this work we studied the effects of KIAA1199 knockdown for the first time *in vivo.* We demonstrated the inhibition in tumor incidence and growth rate. Our findings are in concordance with the results of the proteomic study where we observed modulation of several proteins involved in cell cycle progression and division such as ANAPC10 (Anaphase-promoting complex subunit 10), PPP1CB (Serine/threonine-protein phosphatase PP1-beta protein catalytic subunit) and PPP2R1A (Serine/threonine-protein phosphatase 2A regulatory subunit) upon KIAA1199 knockdown. All of these proteins play role in cell cycle regulation and cell division. For example ANAPC10 participates in the progression through mitosis and the G1 phase of the cell cycle [38]. PPP1CB is a component of the PTW/PP1 phosphatase complex, which plays a role in the control of chromatin structure and cell cycle progression during the transition from mitosis into interphase [39] and PPP2R1A is required for proper chromosome segregation and for centromeric localization in mitosis [40]. These data suggest an important role for KIAA1199 in breast cancer incidence, growth and progression.

Mass spectrometry based proteomics holds special promise to provide better insights into biological pathways. In this study, we pursued the functional analysis of KIAA1199 in breast cancer cells as a novel target screened in our previous proteomic study [3]. Although the detailed mechanism of KIAA1199-mediated cellular responses is still obscure, our proteomic study shed light on how different biological pathways may be influenced by KIAA1199 directly or indirectly. For instance alteration of components of MAPK, NF-k-B and apoptosis pathways can potentially affect other cellular phenomena such as angiogenesis.

Furthermore, our findings suggest that KIAA1199 knockdown may also affect the cellular metabolism. It is known that tumor cells typically have much higher rates of glycolysis compared to their normal tissues of origin; consequently they secrete glucose-derived carbon mostly as lactate instead of completely oxidizing glucose. This phenomenon is known as the Warburg effect [41,42]. In this study we observed the modulation of several metabolism associated enzymes. The KIAA1199 knockdown cells have lower expression of proteins involved in glycolysis and cytosolic break down of glucose (such as PGAM4) and instead tend to the mitochondrial oxidation. Therefore, the Warburg effect which is a fundamental character of cancer cells also seems to be negatively influenced by KIAA1199 depletion.

We utilized various approaches and techniques to comprehensively evaluate the major consequences of KIAA1199 depletion in breast cancer cells *in vitro* and *in vivo*. Despite the limitations in study sizes (the number of TMA tissues, animals, cell lines etc.) we studied several aspects of cancer development and progression following KIAA1199 knockdown. Further studies on each aspect with larger sample sizes will help to uncover the mechanism of KIAA1199 function and provide more evidences. Taken together, our findings presented here suggest that KIAA1199 may represent a novel target for biomarker development and a novel therapeutic target to control breast cancer progression and metastasis.

## Conclusions

Our TMA/IHC study confirmed the results of bioinformatics studies from a large database of microarray analyses which show the overexpression of KIAA1199 in breast carcinoma. We showed *in vitro* the inhibition of cell proliferation and migration as well as apoptosis enhancement in MDA-MB-231 cells upon KIAA1199 knockdown. Silencing of KIAA1199 resulted in decreased tumor incidence and tumor growth rate *in vivo*. Our proteomic analysis provided insight into the pathways through which KIAA1199 may affect a broad range of cellular functions including apoptosis, metabolism and cell motility.

## Abbreviations

IHC: Immunohistochemistry; TMA: Tissue microarray; LC-MS/MS: Liquid chromatography tandem mass spectrometry; SCX: Strong cation exchange; SILAC: Stable isotope labeling by amino acids in cell culture.

## Competing interests

The authors declare that they have no competing interests.

## Authors’ contributions

SJD designed the research; MSJ, JH, YL, LG, JD and JW carried out the vector construction and stable cell line generation. MSJ, JH, and HP carried out the Western blotting. MSJ carried out the RT-PCR. MSJ, JH and MLV carried out the cell cycle, apoptosis, motility, migration, and proliferation assays. MSJ, MLV and HH carried out the tumor growth, IHC, and TMA/IHC. MSJ, JH, ML and XH carried out the SILAC based quantitative proteomic study. MSJ, XH, KF, and FY carried out the data analysis; XH and FY carried out the statistical analysis. MSJ carried out the interpretation of proteomic results. SJD and RKS. led the research; MSJ, and SJD wrote the paper. All of the authors have been involved in revising the manuscript and have given final approval of the version to be published.

## Pre-publication history

The pre-publication history for this paper can be accessed here:

http://www.biomedcentral.com/1471-2407/14/194/prepub

## Supplementary Material

Additional file 1: Table S1 The sequence of the primers used in this study for the semi-quantitative RT-PCR analysis. Click here for file

Additional file 2: Figure S1 A) The Olfactory Bulb tissue was used as negative control tissue for KIAA1199 staining. B) The human kidney tissue was used as both positive (cells in tubules) and negative (cells in glomeruli) control tissues for immunohistochemical staining (according to the human protein atlas at http://www.proteinatlas.org KIAA1199 has the highest expression level in renal tubules). C) Technical negative control staining (without primary antibody) for the human kidney tissue. D) Higher magnification of stained kidney tissue (×200 magnifications) shows the cytosolic localization of KIAA1199 in positive cells (renal tubules). Click here for file

Additional file 3: Figure S2 Knockdown of KIAA1199 in MDA-MB-231 cells. A) The empty pGPH1/GFP/NEO shRNA expression vector used to generate MDA-MB-231-ShNC and Hs578T-ShNC cells. B) The sequence of two different KIAA1199 specific inserts which were used to generate the MDA-MB-231-ShA, MDA-MB-231-ShB, Hs578T-ShA and Hs578T-ShB cell lines. C) Top: RT-PCR analysis shows a dramatic decrease of KIAA1199 mRNA expression in the knockdown cells; the transcript of (glyceraldehyde 3-phosphate dehydrogenase) GAPDH was used as control. Bottom: The bands obtained after the electrophoresis were quantified by densitometry, and their intensity was normalized to that provided by the GAPDH band (relative integral optical density (IOD)). The average of normalized intensity values (triplicate) obtained from the negative controls was set to 100%. D) Top: Western blotting shows a dramatic decrease in KIAA1199 protein in knockdown cells. Bottom: The bands obtained from triplicate experiments were quantified by densitometry, and their intensity was normalized to corresponding replicate of α-Tubulin band (relative integral optical density (IOD)). The average of normalized intensity values (triplicate) obtained from the negative controls was set to 100%. Click here for file

Additional file 4: Table S2 Detailed information about the proteins identified in the SILAC peptide mixture of MDA-MB-231-ShNC cells (L) and MDA-MB-231-ShB cells (H). Click here for file
