# Supplementary figures and images for: Functional proteomic analysis reveals the involvement of KIAA1199 in breast cancer growth, motility and invasiveness
Source: BMC Cancer. 2014 Mar 15;14:194. doi: 10.1186/1471-2407-14-194 (PMC4007601; doi:10.1186/1471-2407-14-194)

A

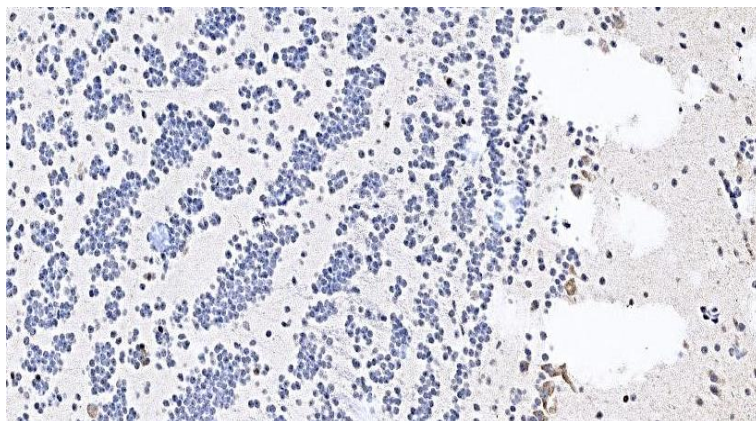

B

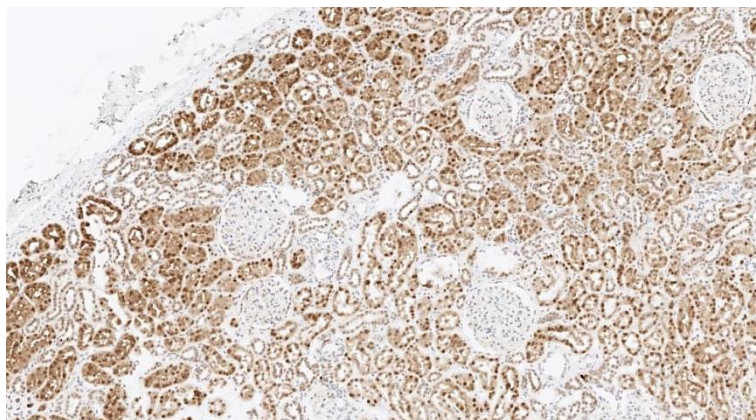

C

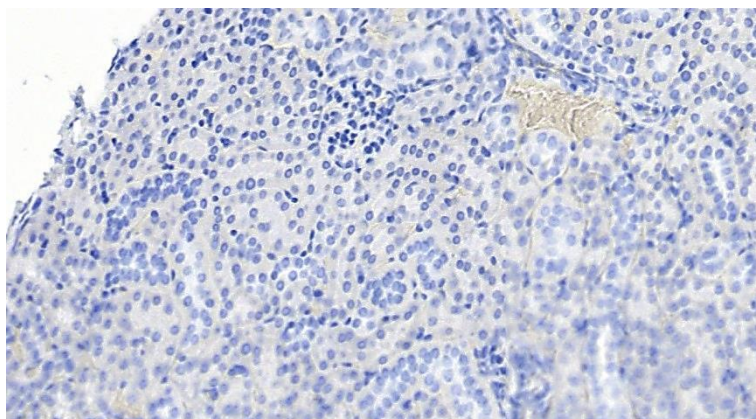

D

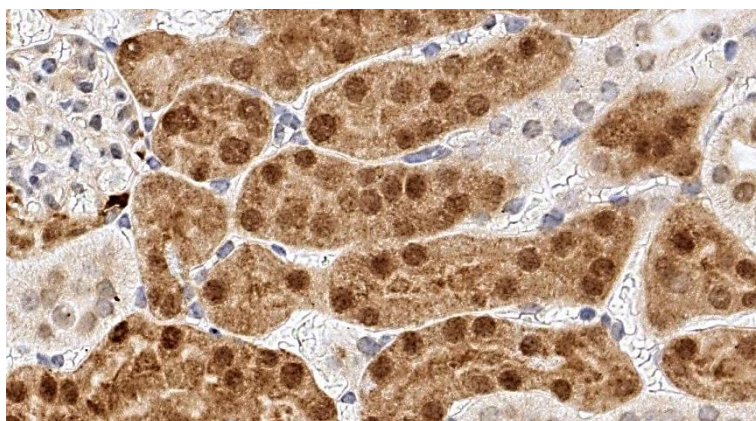

Supplement: Additional file 2: Figure S1 — A) The Olfactory Bulb tissue was used as negative control tissue for KIAA1199 staining. B) The human kidney tissue was used as both positive (cells in tubules) and negative (cells in glomeruli) control tissues for immunohistochemical staining (according to the human protein atlas at http://www.proteinatlas.org KIAA1199 has the highest expression level in renal tubules). C) Technical negative control staining (without primary antibody) for the human kidney tissue. D) Higher magnification of stained kidney tissue (×200 magnifications) shows the cytosolic localization of KIAA1199 in positive cells (renal tubules). [file 1471-2407-14-194-S2.pdf]
