# Supplementary material for: Functional proteomic analysis reveals the involvement of KIAA1199 in breast cancer growth, motility and invasiveness
Source: BMC Cancer. 2014 Mar 15;14:194. doi: 10.1186/1471-2407-14-194 (PMC4007601; doi:10.1186/1471-2407-14-194)
Supplement: Additional file 3: Figure S2 — Knockdown of KIAA1199 in MDA-MB-231 cells. A) The empty pGPH1/GFP/NEO shRNA expression vector used to generate MDA-MB-231-ShNC and Hs578T-ShNC cells. B) The sequence of two different KIAA1199 specific inserts which were used to generate the MDA-MB-231-ShA, MDA-MB-231-ShB, Hs578T-ShA and Hs578T-ShB cell lines. C) Top: RT-PCR analysis shows a dramatic decrease of KIAA1199 mRNA expression in the knockdown cells; the transcript of (glyceraldehyde 3-phosphate dehydrogenase) GAPDH was used as control. Bottom: The bands obtained after the electrophoresis were quantified by densitometry, and their intensity was normalized to that provided by the GAPDH band (relative integral optical density (IOD)). The average of normalized intensity values (triplicate) obtained from the negative controls was set to 100%. D) Top: Western blotting shows a dramatic decrease in KIAA1199 protein in knockdown cells. Bottom: The bands obtained from triplicate experiments were quantified by densitometry, and their intensity was normalized to corresponding replicate of α-Tubulin band (relative integral optical density (IOD)). The average of normalized intensity values (triplicate) obtained from the negative controls was set to 100%. [file 1471-2407-14-194-S3.pdf]

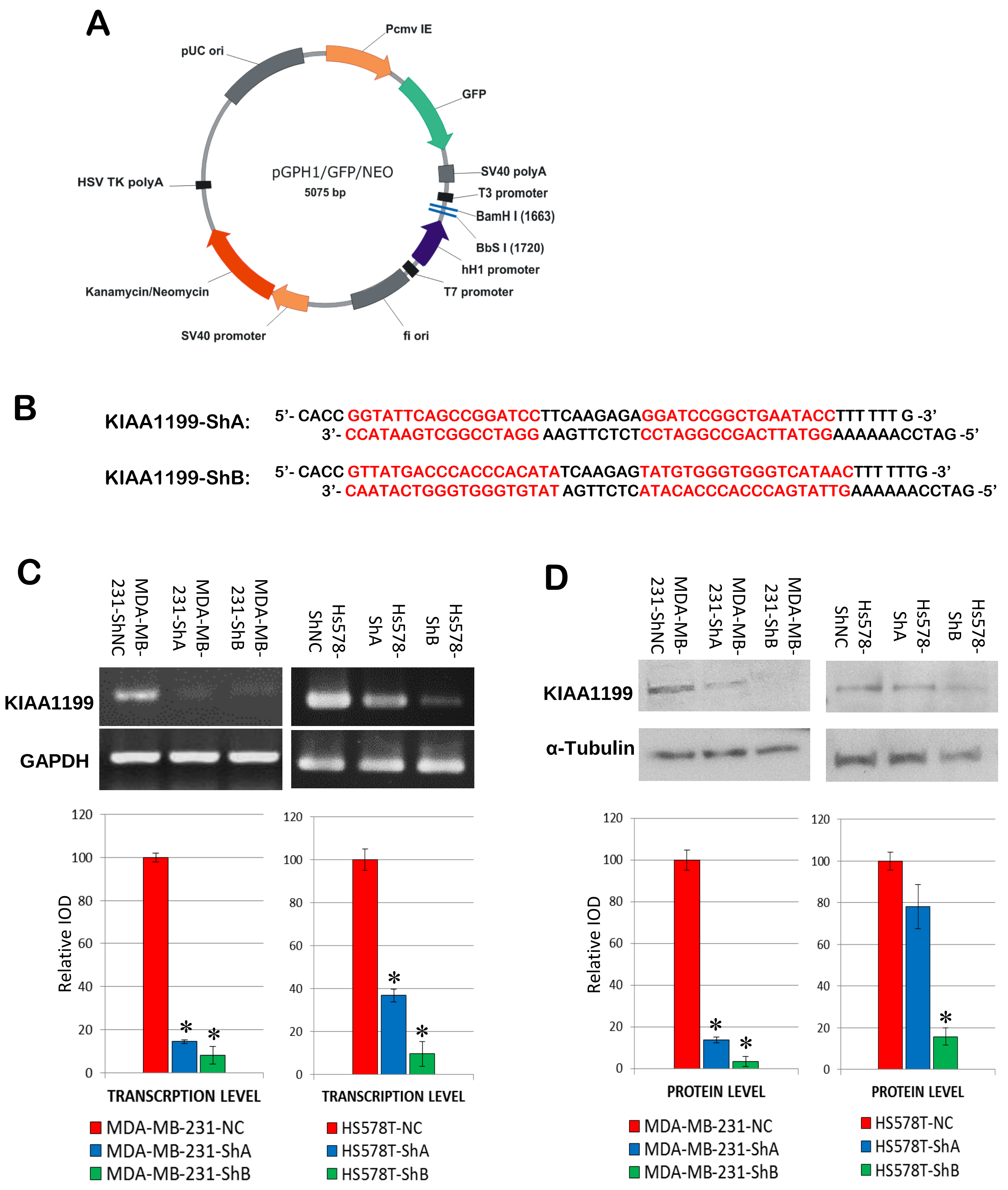

A
pGPH1/GFP/NEO
5075 bp
Pcmv IE
GFP
SV40 polyA
T3 promoter
BamH I (1663)
BbS I (1720)
hH1 promoter
T7 promoter
fi ori
SV40 promoter
Kanamycin/Neomycin
HSV TK polyA
pUC ori
B
KIAA1199-ShA:
5’- CACC GGTATTCAGCCGGATCCTTCAAGAGAGGATCCGGCTGAATACCTTT TTT G -3’
3’- CCATAAGTCGGCCTAGG AAGTTCTCTCCTAGGCCGACTTATGGAAAAAACCTAG -5’
KIAA1199-ShB:
5’- CACC GTTATGACCCACCCACATATCAAGAGTATGTGGGTGGGTCATAACTTT TTTG -3’
3’- CAATACTGGGTGGGTGTAT AGTTCTCATACACCCACCCAGTATTGAAAAAACCTAG -5’
C
MDA-MB-231-ShNC
MDA-MB-231-ShA
MDA-MB-231-ShB
Hs578-ShNC
Hs578-ShA
Hs578-ShB
KIAA1199
GAPDH
Relative IOD
TRANSCRPTION LEVEL
TRANSCRIPTION LEVEL
MDA-MB-231-NC
MDA-MB-231-ShA
MDA-MB-231-ShB
HS578T-NC
HS578T-ShA
HS578T-ShB
D
MDA-MB-231-ShNC
MDA-MB-231-ShA
MDA-MB-231-ShB
Hs578-ShNC
Hs578-ShA
Hs578-ShB
KIAA1199
α-Tubulin
Relative IOD
PROTEIN LEVEL
PROTEIN LEVEL
MDA-MB-231-NC
MDA-MB-231-ShA
MDA-MB-231-ShB
HS578T-NC
HS578T-ShA
HS578T-ShB
